# Sublinear information bottleneck based two-stage deep learning approach to genealogy layout recognition

**DOI:** 10.3389/fnins.2023.1230786

**Published:** 2023-06-30

**Authors:** Jianing You, Qing Wang

**Affiliations:** College of Information and Electrical Engineering, China Agricultural University, Beijing, China

**Keywords:** genealogy layout recognition, sublinear information bottleneck, YOLOv5 detector, ResNet, deep learning

## Abstract

As an important part of human cultural heritage, the recognition of genealogy layout is of great significance for genealogy research and preservation. This paper proposes a novel method for genealogy layout recognition using our introduced sublinear information bottleneck (SIB) and two-stage deep learning approach. We first proposed an SIB for extracting relevant features from the input image, and then uses the deep learning classifier SIB-ResNet and object detector SIB-YOLOv5 to identify and localize different components of the genealogy layout. The proposed method is evaluated on a dataset of genealogy images and achieves promising results, outperforming existing state-of-the-art methods. This work demonstrates the potential of using information bottleneck and deep learning object detection for genealogy layout recognition, which can have applications in genealogy research and preservation.

## 1. Introduction

A genealogy is a special document that records the family lineage and important figures in a family's history in the form of a chart (Wang and Zhang, 2008). It is a characteristic of Chinese civilization, and is a historical record of the bloodline of a kinship group, including the people and events related to the same ancestor. Genealogy is a valuable humanistic resource that plays an irreplaceable and unique role in in-depth research in fields such as history, folklore, demography, sociology, and economics. However, due to wars and social upheavals in history, the lineages and genealogies of many families have been destroyed or lost. Therefore, digital preservation of genealogy has become necessary. Through digital technology, genealogy can be digitally stored and disseminated, making it convenient for researchers and scholars to access and study, and protecting the cultural value and inheritance of genealogy (Chang, 2014).

Genealogy recognition technology is one of the important means of digital preservation of genealogy. By recognizing the ancient books of genealogy, the information in the genealogy can be automatically extracted and processed, thus realizing the digital storage and dissemination of genealogy (Fan, 2013). However, due to the complex and diverse layout structures of genealogy ancient books, the recognition difficulty is also high. Therefore, accurate layout detection and positioning technology is an important prerequisite for genealogy recognition.

Genealogy recognition relies heavily on document layout analysis technology. The most famous and widely used traditional document layout analysis algorithm is the Docstrum algorithm proposed by L. (1993). It sequentially divides the black and white connected domains in the image into text, text lines, and text blocks from bottom to top, thus obtaining the layout. For table recognition, the table lines are obtained through erosion, dilation, and other operations, the row and column areas are divided, and then the cells are combined with text contents to reconstruct the table object. In response to some shortcomings of the algorithm, subsequent researchers have proposed corresponding optimization algorithms. For example, Wieser and Pinz (1994) proposed a method of combining bottom-up merging and top-down cutting for newspaper page segmentation. Watanabe et al. (1995) introduced a classification tree to manage different types of layout structures, and proposed a method for recognizing the layout structure of documents with multiple table formats. Liu-Gong et al. (1995) used a universal model to convert document images into layout structures. Lee et al. (2000) used geometric structure analysis to propose a knowledge-based method for analyzing complex geometric structures of journal pages. Lee and Ryu (2001) constructed a pyramid quadtree structure for multiscale analysis based on a parameter-free method, and proposed a periodicity measurement method to find the periodic properties of text regions. In addition, in the application of textual literature, Bukhari et al. (2011) proposed a layout distribution system for extracting text in reading order from scanned images of Arabic texts written in different languages and styles. However, traditional algorithms still face some technical challenges, mainly in (1) layout analysis and table structure extraction; (2) image processing methods relying on various threshold and parameter selections; (3) difficulty in ensuring generalization of document images in different scenarios.

In recent years, deep learning has shown great promise in improving the accuracy and efficiency of genealogy layout recognition. With its ability to automatically learn and extract features from large datasets, deep learning models can also adapt to different variations in genealogy images, such as variations in font styles, sizes, and orientations, without the need for manual feature engineering (Li et al., 2022). However, there are still some challenges that need to be addressed to improve the performance of deep learning models in genealogy recognition. One of the major challenges is dealing with images that are poorly scanned or contain noise or artifacts, which can affect the accuracy of recognition. Another challenge is handling complex layouts, which can make it difficult for deep learning models to distinguish between text and non-text regions. Moreover, the lack of large and diverse annotated datasets for genealogy recognition limits the performance of deep learning models. This is because deep learning models require large amounts of annotated data to effectively learn and generalize to new data. Therefore, efforts to create more annotated datasets for genealogy recognition are needed to improve the performance of deep learning models in this domain.

Therefore, in this paper, we present a two-stage deep learning approach for genealogy image layout recognition. Firstly, genealogy images are fed into the ResNet classifier model to identify whether the image contains a bordered or borderless image. Based on the classification results, the image is then directed to either a borderless or a bordered YOLOv5 object detection model. Both the deep learning classifier and object detection models are trained using large amounts of labeled data, which can lead to high accuracy in recognizing different image features and layouts, and can be easily scaled to recognize a large number of different image layouts, making them suitable for recognizing different genealogy image layouts. Further, the proposed method can also be relatively fast at recognizing image layouts, making them suitable for real-time or near real-time applications. Furthermore, we introduce the sublinear information bottleneck (SIB) algorithm to compress the intermediate feature representation of the network model as much as possible while ensuring the accuracy of the model's output, thus achieving high generalization and strong robustness in layout recognition. The main contributions of this paper can be summarized as follows:

(1) Proposing a two-stage deep learning approach that providing advantages of high accuracy, scalability, flexibility, and speed performance;

(2) Introducing the SIB compression technology to improve the network's generalization performance, making it more adaptable to genealogy images.

(3) Using rich collection resources, we scan and manually label common genealogy images to establish a genealogy image and layout positioning standard dataset for model training and experimental result testing.

The rest of this paper is organized as follows: Section 2 reviews related work on deep learning-based layout recognition and the basic concept of information bottleneck. In Section 3, we introduce our proposed layout recognition method in detail, including the SIB, SIB-ResNet classifier and SIB-YOLOv5 object detector. In Section 4, we construct a genealogy layout dataset that we designed and established independently and perform performance testing on our proposed method. Finally, Section 5 summarizes the whole paper.

## 2. Related works

### 2.1. Deep learning based genealogy layout recognition

In recent years, deep learning-based approaches have become increasingly popular for genealogy layout recognition due to their ability to automatically learn discriminative features from data. Borges Oliveira and Viana (2017) proposed a fast automatic document layout method based on convolutional neural networks (CNN), which greatly improved overall performance. Moreover, Kosaraju et al. (2019) proposed a texture-based convolutional neural network model called DoT-Net, which can effectively recognize document component blocks such as text, images, tables, mathematical expressions, and line graphs, solving the problems caused by location transformations, inter-class and intra-class variations, and background noise. Singh and Karayev (2021) unveil an architecture for a Handwritten Text Recognition (HTR) model based on Neural Networks, which is capable of recognizing complete pages of handwritten or printed text without the need for image segmentation. It is built on the Image to Sequence architecture, allowing it to accurately extract text from an image and sequence it correctly. Additionally, it can be trained to generate auxiliary markup that pertains to formatting, layout, and content. Wu et al. (2023) proposed a genealogical knowledge graph model to implement the construction and applications of genealogical knowledge graphs. One of the challenges in genealogy layout recognition is the lack of large datasets, as well as the presence of various types of noise, such as text overlap, low contrast, and curved text. To address this, researchers have proposed different strategies, such as dataset collection and data augmentation.

In Singh and Karayev (2021), Sumeet et al. presented TableBank, a new image-based table detection and recognition datase with 417K high quality labeled tables, allowing building strong baselines of deep neural networks. Zhong et al. (2019) introduced the PubLayNet dataset for document layout analysis, the dataset is created by automatically associating the XML representations with the content of more than 1 million publicly available PDF articles on PubMed Central. Data augmentation is also a commonly used technique to increase the size and diversity of training data. To address the issue of data scarcity for rare family relationships, He et al. (2021) leveraged data augmentation technology to generate additional synthetic data. Subsequently, they developed a multitask-based artificial neural network model capable of simultaneously detecting names, extracting relationships between individuals, and assigning attributes such as birth and death dates, residence, age, and gender.

Deep learning-based approaches have shown promising results for genealogy layout recognition. However, there is still room for improvement, particularly in handling complex genealogy layouts with overlapping and curved text.

### 2.2. Information bottleneck

Information Bottleneck (IB) was first proposed by Tishby and Zaslavsky (1999) for traditional machine learning methods. In 2015, Tishby hypothesized in his paper that deep learning is an information bottlenecking procedure that compresses data noise as much as possible and keeps the information that the data wants to convey (Tishby and Zaslavsky, 2015). This suggests that neural networks are like squeezing information into a bottleneck, leaving only the features that are most relevant to the general concept and removing the large amount of irrelevant and noisy data. Later, it was used in Schwartz-Ziv and Tishby (2017) for the study of interpretability of deep learning, and realized the effective combination of information bottleneck theory and deep learning networks.

The Information Bottleneck is an information theory method used for tasks such as data compression and classification, which effectively extracts key information from data. The core idea is to minimize the uncertainty of the output information while retaining the maximum amount of input information.

Specifically, given input random variable *X* and output random variable *Y*, the IB method finds an intermediate random variable *T* to describe the relationship between input and output, which maximally preserves the information of *X*, while minimizing the information entropy between *T* and *Y*, i.e.,


(1) $$\begin{array}{l} I\left(T;X\right)-\beta I\left(T;Y\right) \end{array}$$

where *I*(*T*; *X*) and *I*(*T*; *Y*) are the mutual information between *T* and *X*, and *T* and *Y*, respectively, and β is a tuning parameter that balances the information entropy between *X* and *Y*, and that between *T* and *Y*.

The advantage of the IB method is that it can automatically learn the most important features from data without requiring prior knowledge, thus extracting the most useful information. It has been widely used in natural language processing, image recognition, signal processing, and other fields.

Dong and He (2023) used the optimization objective proposed by the information bottleneck theory, added a loss function to the tensor input to the linear classification layer in the model, and aligned the clean samples with the high-level features obtained when the adversarial samples are input to the model by sample cross-training. Li and Liu (2019) employed IB theory to understand the dynamic behavior of convolutional neural networks (CNNs) and investigate how the fundamental features have impact on the performance of CNNs. To construct a classifier which is more robust to small perturbations in the input space, Pensia et al. (2020) propose a novel strategy for extracting features in supervised learning. The experimental results for synthetic and real data sets show that the proposed feature extraction methods indeed produce classifiers with increased robustness to perturbations. In Song et al. (2022), Song et al. investigated for the first time a novel and flexible multimodal representation learning method, multi-feature deep information bottleneck (MDIB), for breast cancer classification in CESM. Moreover, Amjad and Geiger (2020) investigate training deep neural networks (DNNs) for classification via minimizing the information bottleneck (IB) functional. The above studies show that information bottleneck theory has a positive impact on feature extraction, model optimization and performance improvement of deep learning models.

## 3. Proposed method

### 3.1. Sublinear information bottleneck

The SIB is a novel method for unsupervised feature selection and compression that extends the original information bottleneck (IB) method by incorporating second-order statistics of the input data (Kolchinsky et al., 2018).

The SIB method aims to find a compressed representation *T* of the input data *X* that preserves the most relevant information for a given task *Y*. Specifically, the method seeks to minimize the following objective function:


(2) $$\begin{array}{l} L=I^{2}\left(X;T\right)+\beta H\left(Y|T\right) \end{array}$$

where *I*^2^(*X*; *T*) is the second-order mutual information between *X* and *T*, and *H*(*Y*|*T*) is the conditional entropy of *Y* given *T*.

The first term *I*^2^(*X*; *T*) measures the amount of second-order statistical dependence between *X* and *T*, while the second term *H*(*Y*|*T*) measures the amount of uncertainty in predicting *Y* given *T*. By minimizing this objective function L, the SIB method finds a compressed representation *T* that preserves the most relevant information for predicting *Y*.

The optimization problem is typically solved using a Lagrangian relaxation approach, which leads to a set of non-linear equations that can be solved iteratively (Juttner et al., 2001). Compared to the original IB method (Owen, 2001), the SIB method takes into account the second-order statistics of the input data, which can capture higher-order dependencies and correlations between input variables. This can lead to better feature selection and compression performance, especially in complex datasets with non-linear dependencies.

The conventional problem in IB theory is to minimize the mutual information *I*(*X*; *T*) with respect to the encoding mapping *p*(*t*|*x*), given a fixed input distribution *p*(*x*). This function is convex. On the other hand, maximizing the conditional entropy *H*(*Y*|*T*) = *H*(*Y*)−*I*(*T*; *Y*) with respect to the decoding mapping *p*(*y*|*t*), given a fixed joint distribution *p*(*x, y*), is a concave function. The entropy *H*(*Y*) is a constant for the dataset or assumed to have a small fluctuation for a patch of training data. Therefore, the SIB function L used to determine the global or local minimum is a concave function.

We define the optimal parameter set ω to achieve the best performance of the minimal value of the loss L and a randomly generated parameter set ϕ. Let the representation predicted by ϕ be Ŷ, and ŷ be an instance of Ŷ. Thus, we have


(3) $$\begin{array}{l} H\left(q_{\phi }\left(Y|T\right)\right)\leq H\left(q_{\phi }\left(Y|T\right)\right)+D_{KL}\left(q_{\omega }\left(Y|T\right)∥q_{\phi }\left(Y|T\right)\right)\\ =-{\text{E}}_{q_{\omega \left(Y,T\right)}}\left[\text{log}q_{\phi }\left(Y|T\right)\right]\\ =-{\text{E}}_{q_{\omega \left(Y,Ŷ\right)}}\left({\text{E}}_{q_{\phi \left(Ŷ,T\right)}}\left[\text{log}q_{\phi }\left(Y|Ŷ\right)\right]\right)\\ =-{\text{E}}_{q_{\omega \left(Y,Ŷ\right)}}\left[\text{log}q_{\phi }\left(Y|Ŷ\right)\right]\\ =C\left(q_{\phi }\left(Y|Ŷ\right)\right) \end{array}$$

where **E**· denotes the expectation value, C(·) is the cross-entropy, and (a) is due to


(4) $$\begin{array}{l} q_{Y|T}\left(y|t\right)=q_{Y|q\left(ŷ|t\right)}\left(y|q\left(ŷ|t\right)\right)=q_{Y|Ŷ}\left(y|ŷ\right) \end{array}$$

The equality in (3) is achieved only when *q*_ϕ_(*y*|*t*) is identical to the optimal mapping *q*_ω_(*y*|*t*). Moreover, if the Kullback-Leibler (KL) divergence *D*_*KL*_(*q*_ω_(*Y*|*T*)∥*q*_ϕ_(*Y*|*T*)) → 0, then *H*(*q*_ϕ_(*Y*|*T*)) → C(*q*_ϕ_(*Y*|Ŷ)). This implies that minimizing the distance between the network parameter set ϕ and the optimal set ω leads to a smaller gap between *H*(*q*_ϕ_(*Y*|*T*)) and its upper bound. The term *I*(*X*; *T*) denotes the information that is compressed from the input signal *X* to the intermediate activation *T*:


(5) $$\begin{array}{ll} I\left(X;T\right)=\underset{x,t}{\sum }q\left(x,t\right)\log\left(\frac{q\left(x,t\right)}{p\left(x\right)q\left(t\right)}\right)\; & \;\\ =\underset{x,t}{\sum }q\left(x,t\right)\log\left(\frac{q\left(t|x\right)}{q\left(t\right)}\right)\; & \;\\ =\underset{x,t}{\sum }q\left(x,t\right)\log q\left(t|x\right)-\underset{t}{\sum }q\left(t\right)\log q\left(t\right) \end{array}$$

Computing the marginal distribution of *T*, $q\left(t\right)=\underset{x}{\sum }q\left(t|x\right)p\left(x\right)$, may pose a challenge. Taking inspiration from VIB (Alemi et al., 2017), we employed the variational distribution *r*(*t*) to approximate *q*(*t*). As the KL divergence is non-negative by definition, we obtain:


(6) $$\begin{array}{l} D_{KL}(q\left(T\right)∥r\left(T\right))=\underset{t}{\sum }q\left(t\right)\log q\left(t\right)-\underset{t}{\sum }q\left(t\right)\log r\left(t\right)\\ \geq 0 \end{array}$$

According to (5) and (6), we have


(7) $$\begin{array}{l} I\left(X;T\right)\leq \sum_{x,t}q(x,t)\log q(t|x)-\sum_{t}q(t)\log r(t)\\ \;=\sum_{x,t}p(x)q(t|x)\log q(t|x)-\sum_{x,t}p(x)q(t|x)\log r(t)\\ \;=\frac{1}{N}\sum_{n=1}^{N}q(t|x_{n})\log\frac{q(t|x_{n})}{r(t)}\\ \;=\frac{1}{N}\sum_{n=1}^{N}D_{KL}\left[q\left(T|x_{n}\right)∥r\left(T\right)\right] \end{array}$$

where *N* is the number of data samples that has been defined before.

By combining (3) and (7) with the constraints *H*(*Y*|*T*) ≥ 0, *I*(*X*; *T*) ≥ 0, we established an upper bound for our proposed SIB, which is given by:


(8) $$\begin{array}{l} L\leq \bar{L}=C\left(q_{\phi }\left(Y|Ŷ\right)\right)+\beta [\frac{1}{N}\overset{N}{\underset{n=1}{\sum }}D_{KL}[q\left(T|x_{n}\right)∥r\left(T\right)]{]}^{2} \end{array}$$

The minimization of the loss function L can be transformed into the minimization of the upper bound $\bar{L}$, thereby achieving the objective of reducing L.

### 3.2. SIB-ResNet

**Figure 1:**
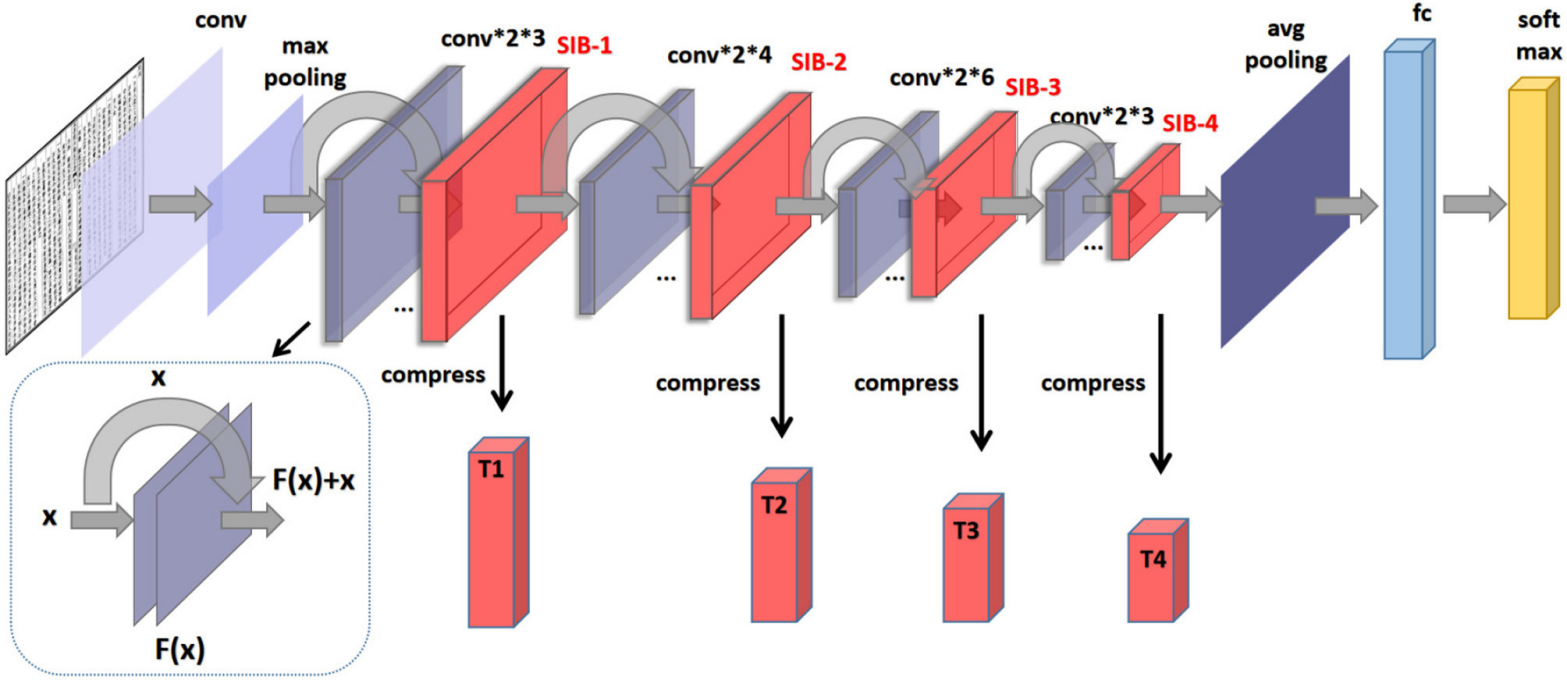
The proposed structure of SIB-ResNet.

In recent years, ResNet has become one of the most popular deep neural network architectures for image classification and other computer vision tasks (He et al., 2016). One of the keys to its success is the use of residual connections, which allow the network to effectively learn features at multiple scales while minimizing the vanishing gradient problem. However, ResNet still suffers from the curse of dimensionality, as the feature maps tend to become increasingly complex and high-dimensional as they pass through the network. To alleviate this issue, we propose to insert four proposed sublinear information bottleneck (SIB) layers into ResNet, which aims to extract the most relevant information from the input while discarding the redundant information. These layers perform dimensionality reduction by encoding the feature maps into a compressed representation, which can then be decoded back to the original dimensionality. By adding these SIB layers, we aim to improve the efficiency and scalability of ResNet, while maintaining its high accuracy. As shown in Figure 1, the constructed loss for SIB-ResNet is


(9) $$\begin{array}{l} {ℒ}_{SIB-ResNet}=I^{2}(X;T_{1})+\sum_{i=1}^{3}\alpha_{i}I\left(T_{i};T_{i+1}\right)+\beta H(Y|T_{4}) \end{array}$$

where α_*i*_ > 0, (*i* = 1, 2, 3) and β > 0 are designed parameters to balance the weight of each term.

### 3.3. SIB-YOLOv5

The YOLOv5 model utilizes several loss functions to optimize its performance during training. These include:

(1) Objectness loss: This function aims to identify whether the object is present or not in a given anchor box. It penalizes false positives or negatives when detecting an object in the anchor box.


(10) $$\begin{array}{l} L_{\text{obj}}=\lambda_{\text{obj}}\overset{S^{2}}{\underset{i\in \text{anchors}}{\sum }}\overset{B}{\underset{j=0}{\sum }}1_{ij}^{obj}{\left({\text{IOU}}_{pre}-{\text{IOU}}_{true}\right)}^{2} \end{array}$$

where *i* denotes the index of anchor boxes, *j* denotes the index of bounding boxes, $1_{ij}^{obj}$ is an indicator function that is equal to 1 if the anchor box *i* is assigned to the ground-truth box *j*, and 0 otherwise. *S* is the grid size, and *B* is the number of predicted bounding boxes each anchor predicts. IOU_*pre*_ represents the intersection over union (IOU) between the predicted bounding box and its assigned ground truth box, while IOU_*true*_ represents the true intersection over union (IOU) between the ground truth box and anchor box.

(2) Classification loss: This function helps to classify the object detected in the anchor box. It computes the cross-entropy between the predicted class probabilities and the true class of the object.


(11) $$\begin{array}{l} L_{\text{cls}}=\lambda_{\text{cls}}\overset{S^{2}}{\underset{i\in \text{anchors}}{\sum }}\overset{B}{\underset{j=0}{\sum }}1_{ij}^{obj}\underset{c\in C}{\sum }\\ \left[p_{ij}^{c}\log\left({\hat{p}}_{ij}^{c}\right)+\left(1-p_{ij}^{c}\right)\log\left(1-{\hat{p}}_{ij}^{c}\right)\right] \end{array}$$

where $p_{ij}^{c}$ is the indicator function for the *j*_*th*_ bounding box in the *i*_*th*_ anchor box having class *c* and ${\hat{p}}_{ij}^{c}$ is the predicted probability for the same class. $1_{ij}^{obj}$ is the same as in the objectness loss.

(3) Localization loss: This function predicts the bounding box of the object in the image. It computes the mean squared error between the predicted box coordinates and the true box coordinates.


(12) $$\begin{array}{l} L_{\text{loc}}=\lambda_{\text{loc}}\overset{S^{2}}{\underset{i\in \text{anchors}}{\sum }}\overset{B}{\underset{j=0}{\sum }}1_{ij}^{obj}\underset{m\in \left\{x,y,w,h\right\}}{\sum }{\left(\sigma_{ij}^{m}\right)}^{2}{\left({\hat{t}}_{ij}^{m}-t_{ij}^{m}\right)}^{2} \end{array}$$

where $\sigma_{ij}^{m}$ is the mask to select the predicted value of *m*, and $t_{ij}^{m}$ is the true value of *m* for the *j*_*th*_ bounding box in the *i*_*th*_ anchor box. ${\hat{t}}_{ij}^{m}$ is the predicted value for the same parameter.

These loss functions work together to optimize the YOLOv5 model during training, allowing it to detect objects accurately and precisely in images.

The CBL module is a key component of the YOLOv5 object detection model, it performs point-wise linear transformations coupled with pointwise activation functions on a subset of feature maps. This approach reduces the channel dimension input, greatly enhancing the non-linear characteristics of CNN features and improving their descriptive power. The resulting reduction of model complexity lowers computation costs while preserving significant spatial granularity. Compared to YOLOv4 (Bochkovskiy et al., 2020), in YOLOv5, the CBL module has been further improved by introducing a Swish activation function. This activation function is known to provide non-linearity superior to ReLU, making it a popular choice. The CBL module's mathematical formulation is then as follows:


(13) $$\begin{array}{l} y=\text{CBL}\left(x\right)=\sigma \left(\gamma \left(x+\beta \right)\right)\times \text{Swish}\left(x\right) \end{array}$$

Here, *x* is the input tensor, the bias shift β, and scaling parameter γ are learnable parameters, while σ and Swish denote the sigmoid and Swish activation functions, respectively. The YOLOv5 model also makes use of a similar but more complex CBL block, which leverages the power of skip connections to perform deep feature fusion, reducing computation costs and enhancing the model's accuracy simultaneously.

**Figure 2:**
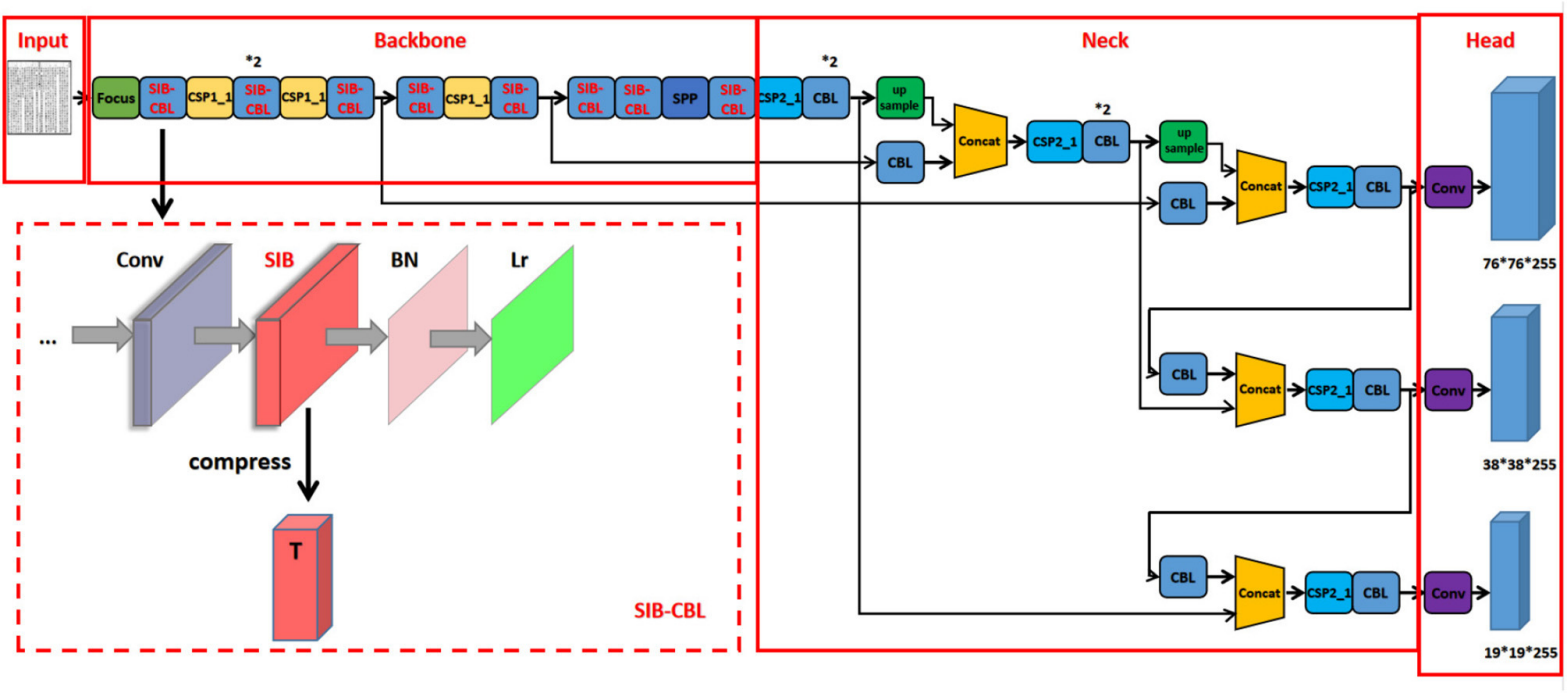
The proposed structure of SIB-YOLOv5. ^*^Represents the number of times the module is repeated in the graph.

As shown in Figure 2, the SIB layer is embedded into CBL module to reduce the dimensions of the CBL input, forcing the module to learn a more concise and reduced representation of the input, thus improving its generalization abilities. Define the original input *X* by *T*_0_, the overall loss of our proposed SIB-YOLOv5 can be summarized as


(14) $$\begin{array}{l} L_{SIB-YOLOv5}=\overset{K}{\underset{i=0}{\sum }}\alpha_{i}I^{2}T_{i};T_{i+1}+\lambda_{1}L_{\text{obj}}+\lambda_{2}L_{\text{cls}}+\lambda_{3}L_{\text{loc}} \end{array}$$

where *K* is the number of CBL modules with SIB, α_*i*_ > 0, (*i* = 1, …, *K*) is the ratios of each SIB, λ_1_, λ_2_ and λ_3_ are the term weights of original YOLOv5 loss.

## 4. Experimental results

### 4.1. Genealogy dataset

**Figure 3:**
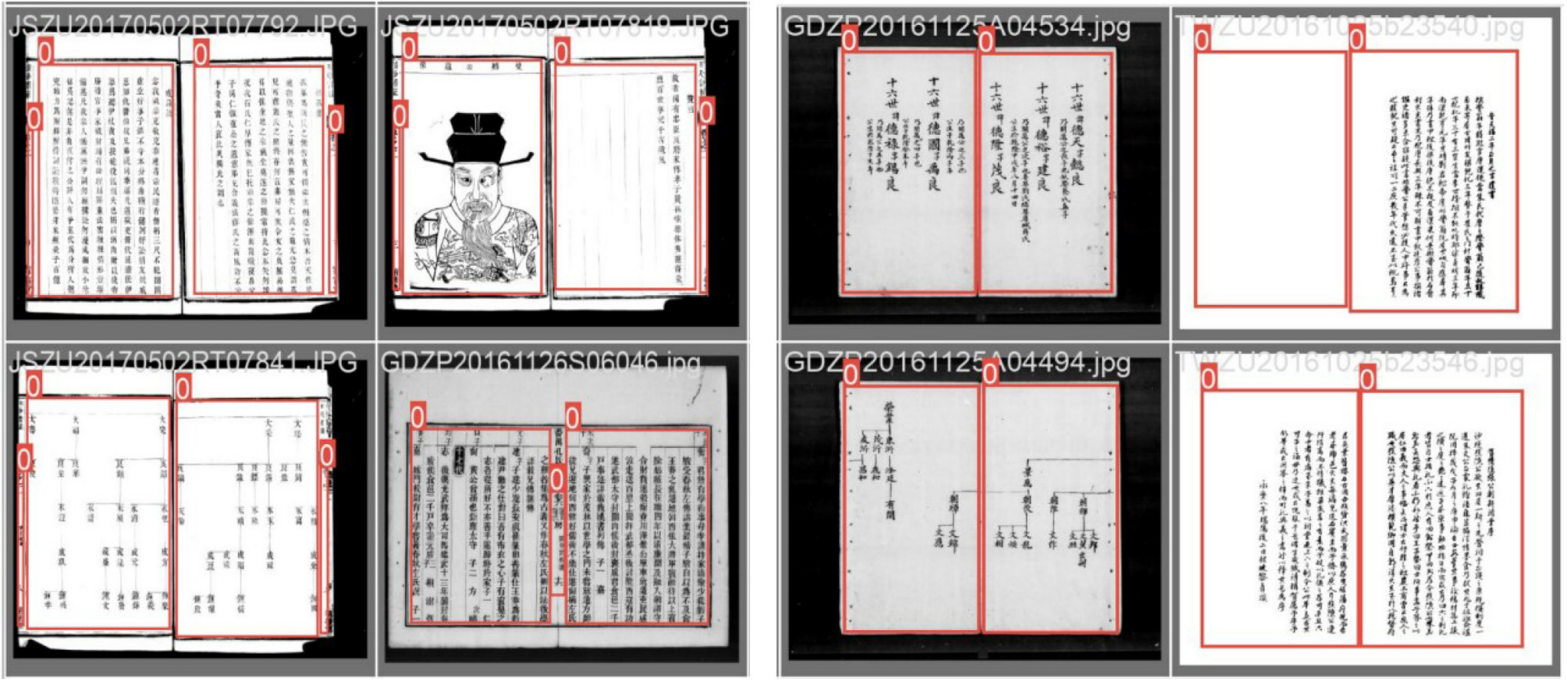
The two styles of genealogical picture and their corresponding tag boxes.

The Chinese genealogy has undergone thousands of years of development, from the undefined format before the Pre-Qin period, to the simple graph format created by Sima Qian, to Ban Gu's four-generation and one-turn format, and then to the graphic transmission and separation format during the Northern and Southern Dynasties. It has gradually improved over time, and even today, new discoveries are still being made in the compilation methodology of Chinese genealogy. There are currently six common types of genealogy samples, and different family tree styles have different effects on the fitting of deep learning models. In this experiment, we mainly scanned and extracted two common styles (with and without borders on the inner pages) and manually labeled the positions of the tag boxes, recording the positions of the upper left and lower right vertices of the tag boxes relative to the images to achieve image labeling. The two styles of genealogical picture and their corresponding tag boxes are shown in Figure 3.

### 4.2. Experimental settings

The experiment was conducted on a server running Ubuntu 20.04 operating system with an Intel(R) Xeon(R) Platinum 8255C CPU and an RTX 3080 GPU with a memory size of 10GB. The training was accelerated using CUDA 11.3, and PyTorch 1.10.0 deep learning framework was used for training. The Resnet classification model and YOLOv5-6.0 prediction model were used, with an input image size of 640 × 640. The initial learning rate was set to 0.01 and the final learning rate was set to 0. The SGD optimizer had a momentum of 0.937, and the batch size for training was set to 16.

**Figure 4:**
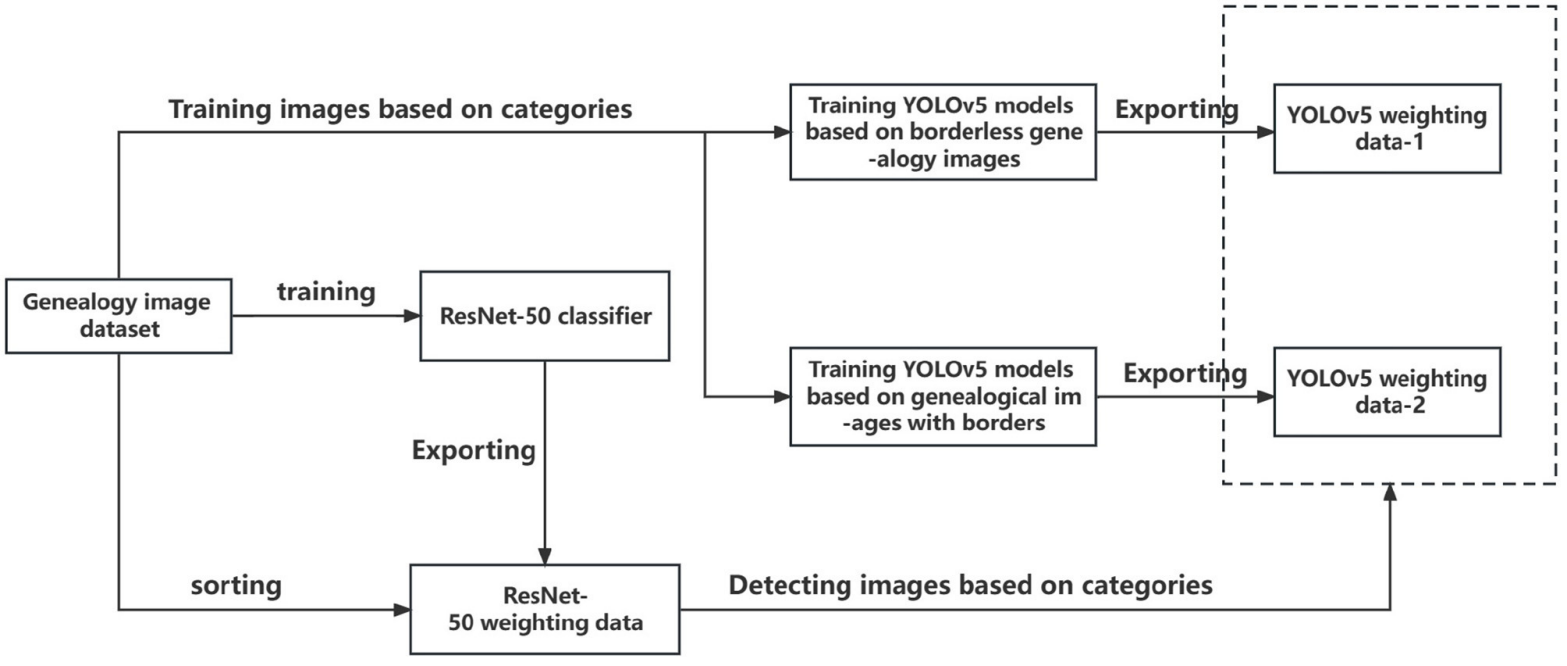
The implementation process of model.

This layout detection method for Chinese genealogy image recognition and region localization, which is divided into two parts: classification and detection. The classification part employs an SIB-ResNet network optimized by the SIB theory for feature extraction, achieving high accuracy. The detection part uses an SIB-YOLOv5 model, where the SIB enhances the model's precision and computational speed compared to the original network. The specific process is shown in the Figure 4. To validate the proposed method, a set of experiments was designed for classification, detection, and overall performance testing.

Experiment 1 compares the classification performance of ResNet, VGGNet, IB-ResNet, and SIB-ResNet through a set of comparative trials. All models were trained on the same data and tested on the same test set to compare their detection speed and accuracy.

Experiment 2 focuses on the detection part and compares the computational efficiency, mAP value of the training set, and accuracy and recall of specific data sets between YOLOv5 and SIB-YOLOv5 models with the same data set and parameter settings during the training process.

Experiment 3 is a comprehensive test that utilizes a specific test set to evaluate the performance of SIB-YOLOv5 models trained on randomly scattered data sets and SIB-YOLOv5 models trained on data sets selected by the SIB-ResNet classifier. The extracted detection areas are compared with the selected positions marked in the local data set, and cosine similarity is calculated. If the similarity reaches the threshold, it is considered a successful recognition. The total number of actual images in the test set, the number of correctly classified images, the number of correct detections, the average completion time, and the overall performance are recorded and evaluated.

### 4.3. Evaluation metrics

This paper evaluates the accuracy of the detection model using the metrics of recall, precision, average precision (AP), and mean average precision (mAP). Before introducing these metrics, the following concepts are defined: TP (true positives) refers to correctly assigned positive samples; TN (true negatives) are the correctly assigned negative samples; FP (false positives) are the incorrectly assigned positive samples; and FN (false negatives) are the incorrectly classified negative samples.

Precision: the proportion of correctly classified positive samples to all samples that the classifier identifies as positive:


(15) $$\begin{array}{l} \text{Precision}=\frac{TP}{TP+FP} \end{array}$$

Recall: the proportion of correctly classified positive samples to all actual positive samples:


(16) $$\begin{array}{l} \text{Recall}=\frac{TP}{TP+FN} \end{array}$$

AP: the area under the curve formed by the precision-recall curve, where recall is on the x-axis and precision is on the y-axis, as shown in (17):


(17) $$\begin{array}{l} \text{AP}=\overset{n-1}{\underset{i=1}{\sum }}\left(r_{i+1}-r_{i}\right)p\left(r_{i+1}\right) \end{array}$$

mAP: the mean average precision of all categories in the dataset, as shown in (18):


(18) $$\begin{array}{l} \text{mAP}=\frac{1}{m}\overset{m}{\underset{i=1}{\sum }}\left(AP_{i}\right) \end{array}$$

### 4.4. Experimental results

In Experiment 1, we compared our proposed SIB-ResNet with ResNet, GooLeNet, VGG, and conventional IB-ResNet, results can be found from Table 1. We can see that, the Resnet network model with the addition of the information bottleneck algorithm is lightweight. SIB-ResNet improves accuracy by nearly 5 percent over the equally lightweight GooLeNet. Compared with the traditional ResNet network, SIB-ResNet obtains faster recognition time while the precision and recall are not affected. SIB-ResNet model has only 4 MB more weights than the IB-ResNet model, and although it is 2 ms slower than IB-ResNet in terms of inference time, the average precision and recall improved by 4.5 and 4.2 percentage points, respectively, over IB-ResNet. VGG is slightly better than SIB-ResNet in terms of average precision and recall, but its weights are about 6 times the weights of SIB-ResNet model, and its inference speed is slightly slower than that of SIB-ResNet.

**Table 1:** Comparison results of Experiment 1.

**Model**	**Weights [MB]**	**mAP [%]**	**Recall [%]**	**Speed [ms]**
ResNet	87.32	96.2	92.5	27
GooLeNet	26.33	90.8	88.9	19
VGG	474.66	97.1	94.8	39
IB-ResNet	74.49	92.3	88.3	22
SIB-ResNet	78.29	96.8	92.5	24

**Figure 5:**
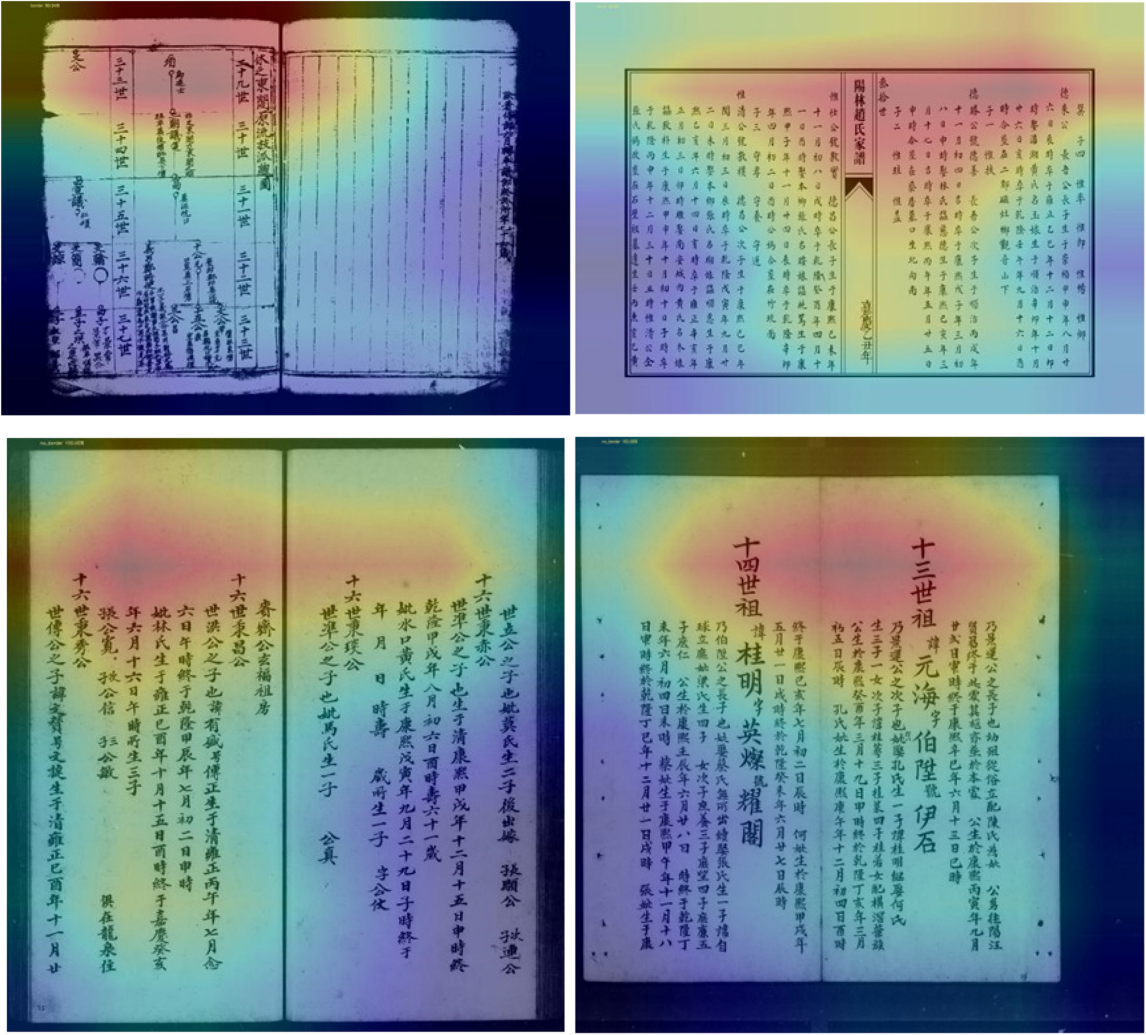
The feature heat map of the SIB-ResNet network.

We also presented the feature heat map of the SIB-ResNet network, as shown in Figure 5, through which we can observe which areas the network focuses on more for the purpose of correct classification. Based on the heat map, we can see that the high response areas are indeed concentrated in the border position area, which is the area that we have identified as the most helpful for making judgments, regardless of whether it is a border or borderless layout.

In Experiment 2, the same data sets and the same parameter settings were used in the training process of both YOLOv5 and SIB-YOLOv5 models to compare the computational efficiency, the mAP values of the training set, and the accuracy and recall of detection for a specific data set of the two different models, and the results were given in Table 2.

**Table 2:** Comparison results of Experiment 2.

**Model**	**Weights [MB]**	**mAP [%]**	**Recall [%]**	**Speed [ms]**
YOLOv5	41.9	90.6	93.7	33
IB-YOLOv5	34.5	87.2	90.8	22
SIB-YOLOv5	35.6	89.2	92.3	24

**Figure 6:**
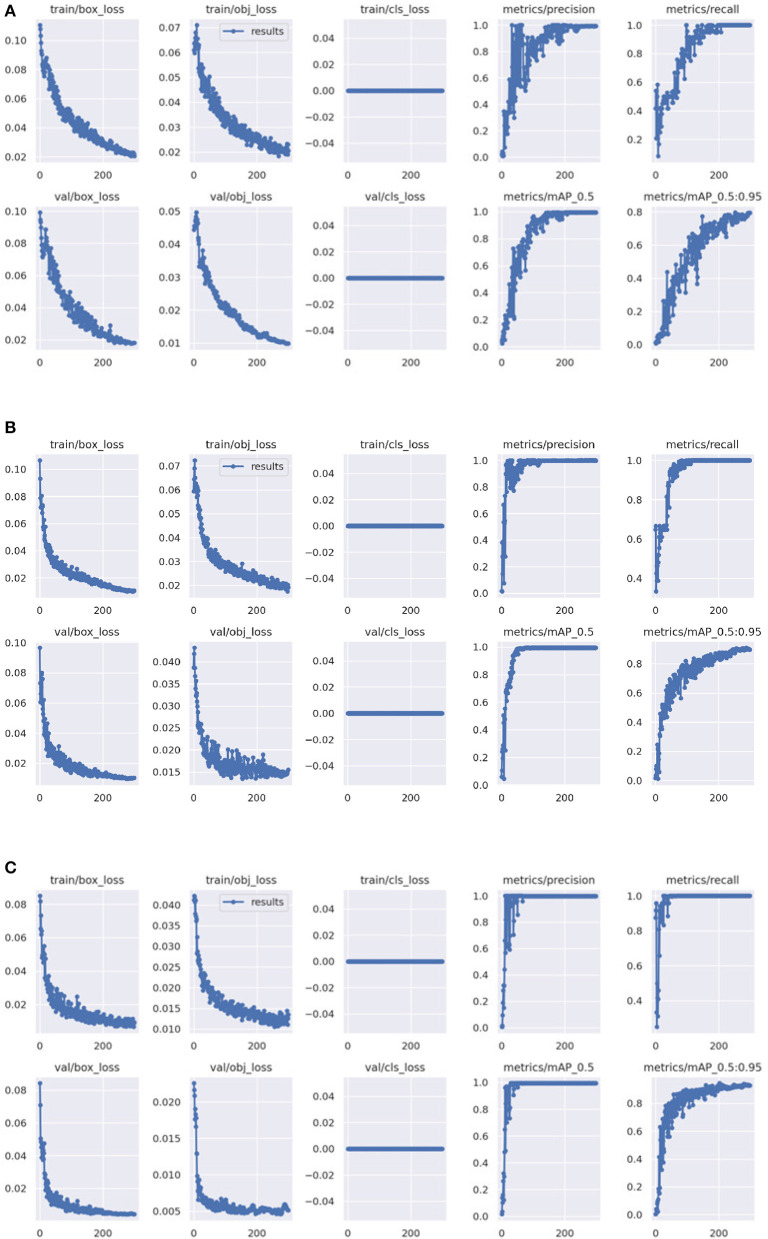
Comparison of the training loss. **(A)** The training loss of YOLOv5. **(B)** The training loss of IB-YOLOv5. **(C)** The training loss of SIB-YOLOv5.

Figure 6 represents the comparison results of various evaluation metrics between YOLOv5 and SIB-YOLOv5. From the figure, it can be seen that the SIB-YOLOv5 model converges faster and has smaller loss values compared to the traditional YOLOv5 model, indicating that training the deep learning network using the introduced sublinear information bottleneck (SIB) improves the convergence ability of the network.

**Figure 7:**
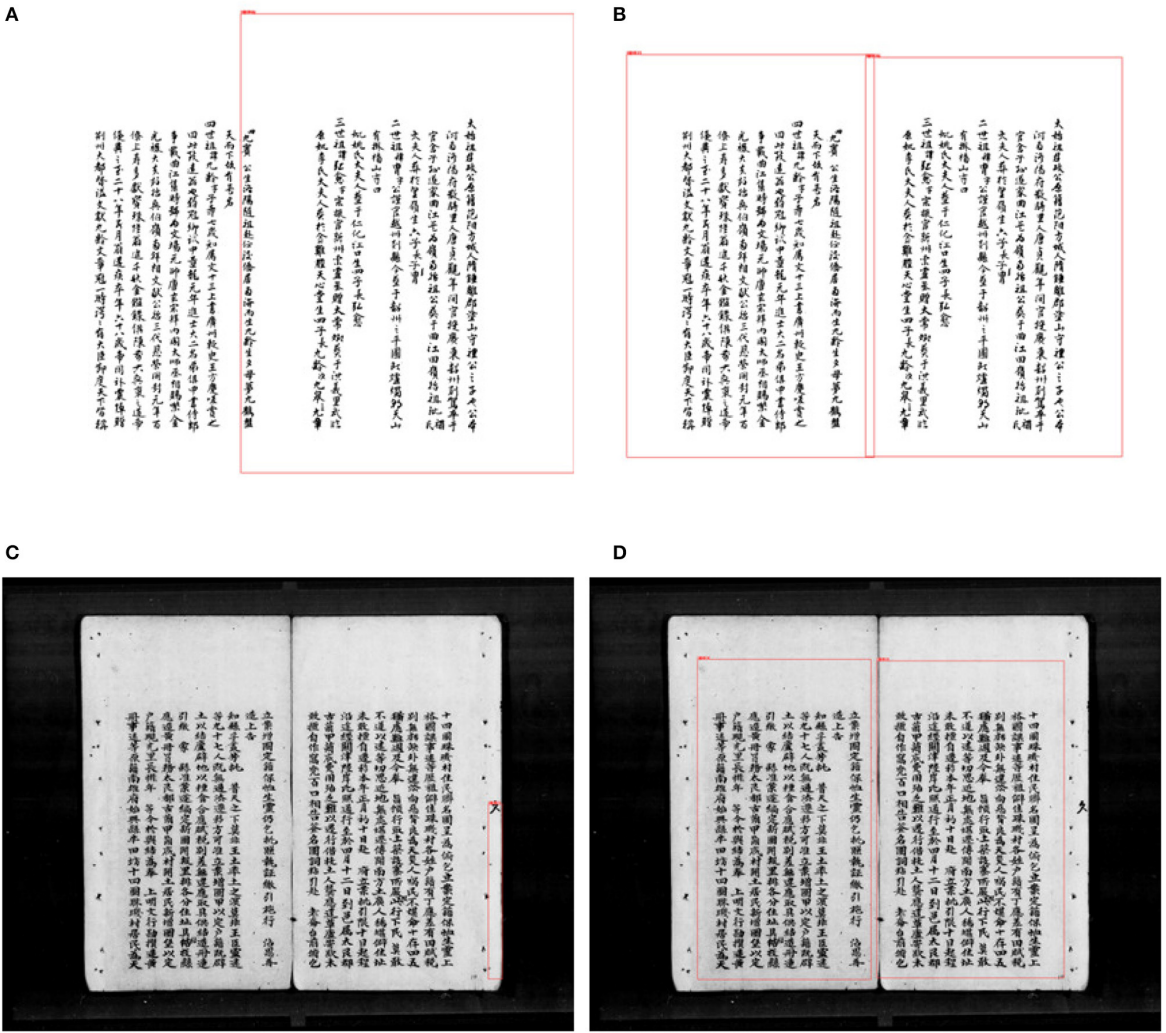
The detection results between the YOLOv5 model trained on the dataset after the SIB-ResNet classifier selection and the YOLOv5 model trained on the randomly selected dataset under different genealogy images. **(A)** Borderless pictures recognized by SIB-YOLOv5. **(B)** Borderless pictures recognized by SIB-YOLOv5 after the SIB-ResNet classifier selection. **(C)** Framed pictures recognized by SIB-YOLOv5. **(D)** Framed pictures recognized by SIB-YOLOv5 after the SIB-ResNet classifier selection.

To better verify the feasibility of the model proposed in this paper, we designed experiment 3 and selected some images of different categories for testing, as shown in Figure 7 for the comparison of the detection results between the YOLOv5 model trained on the dataset after the SIB-ResNet classifier selection and the YOLOv5 model trained on the randomly selected dataset under different genealogy images. Figures 7A, B indicate the detection results for the borderless genealogy images, Figure 7A shows the detection results of the YOLOv5 model trained on the randomly selected dataset, and Figure 7B shows the detection results of the YOLOv5 model trained on the dataset after the SIB-ResNet classifier selection. In the prediction of borderless family tree, the classifier-optimized model is significantly better than the non-classifier-optimized model for the positioning of the borders, and the prediction frames are placed at reasonable locations. Figures 7C, D are the detection result of the genealogy image with border, it can be seen that the detection error of the model with borders mainly comes from the confusion between the borders and text, and the model trained by the classifier can solve this problem well.

Through a series of experiments, we found that the classification of genealogical images using the ResNet34 network has higher training efficiency and recognition accuracy compared to other deep learning networks, and the introduced information bottleneck theory approach enables a lighter and more adaptive model. In the target detection part of Experiment 2, we demonstrate that the SIB-YOLOv5 model shows better performance than the traditional YOLOv5 model, which has poor performance in complex and diverse family tree versions and low target localization accuracy compared to the SIB-YOLOv5 model. The results of Experiment 3 illustrate that the sublinear information bottleneck (SIB) and the two-stage deep learning approach proposed in this paper are robust to complex genealogical picture types, thus showing superior performance as well as more accurate localization accuracy. In the future, as the classifier classifies more and more categories, the model is continuously optimized to be able to perform comprehensive and accurate recognition of genealogical images.

## 5. Conclusion

In this paper, we present a novel sublinear information bottleneck (SIB) approach for genealogy layout recognition and apply it to the ResNet classifier and YOLOv5 object detection network, resulting in the SIB-ResNet and SIB-YOLOv5 models. Compared to traditional IB methods, our SIB is more effective in compressing various types of noise present in genealogy layout images, such as text overlap, low contrast, and stains, while adding minimal additional computational complexity. Our proposed method can simultaneously address the recognition of genealogy layout images with and without borders, demonstrating greater adaptability. Through a series of experimental results, we demonstrate the effectiveness of our approach, achieving excellent performance in both recognition accuracy and computational speed.

## Data availability statement

The raw data supporting the conclusions of this article will be made available by the authors, without undue reservation.

## Author contributions

JY: conceptualization, methodology, validation, formal analysis, writing—original draft preparation, writing—review and editing, and visualization. QW: funding acquisition. All authors have read and agreed to the published version of the manuscript.
